# MicroRNA-378-5p suppresses cell proliferation and induces apoptosis in colorectal cancer cells by targeting BRAF

**DOI:** 10.1186/s12935-015-0192-2

**Published:** 2015-04-15

**Authors:** Zhenlei Wang, Bin Ma, Xiaopin Ji, Yang Deng, Tao Zhang, Xiaojian Zhang, Haoji Gao, Hanxing Sun, Haoxuan Wu, Xianze Chen, Ren Zhao

**Affiliations:** Department of General Surgery, Rui Jin Hospital, Shanghai Jiao Tong University School of Medicine, 197 Rui Jin Er Road, Shanghai, 200025 P R China; Department of Spine, Shanghai East Hospital, Tongji University School of Medicine, Shanghai, 200120 P R China

**Keywords:** Colorectal cancer, miR-378-5p, BRAF, Proliferation, Apoptosis

## Abstract

MicroRNAs (miRNAs) are a group of small non-coding RNA molecules that potentially play a critical role in tumorigenesis. Increasing evidences indicate that miR-378-5p is dysregulated in numerous human cancers including colorectal cancer (CRC) which hypothesizes that miR-378-5p may play an important role in tumorigenesis. However, its role in CRC carcinogenesis remains poorly defined because of lacking target genes information. In the present study, it was demonstrated that the expression of miR-378-5p was down-regulated in CRC tissues and cell lines as determined by quantitative reverse transcription-polymerase chain reaction (qRT-PCR). Furthermore, overexpression of miR-378-5p suppressed cell proliferation, as indicated by CCK-8 assay. Flow cytometric analysis demonstrated that overexpression of miR-378-5p induced cell cycle arrest and promoted apoptosis in CRC cells. A luciferase reporter assay indicated that BRAF was a direct target of miR-378-5p. Western blot and qRT-PCR analysis indicated that BRAF was significantly down-regulated by miR-378-5p in CRC cells. Moreover, miR-378-5p was negatively associated with BRAF in CRC tissues compared to adjacent non-tumor tissues. These results demonstrate that down-regulation of miR-378-5p promotes CRC cells growth by targeting BRAF and restoration of their levels is a potentially promising therapeutic in CRC.

## Background

Colorectal cancer possesses the third highest incidence of human malignant diseases that account for approximately 9.4% of worldwide cancer cases. According to the International Agency for Research on Cancer, about 1 million new cases were detected each year [1]. Much effort has been made on the study of the biological mechanism of CRC and a large number of tumor suppressor genes and oncogenes have been reported recent years. However, the molecular mechanisms underlying the development of CRC are still poorly understood.

miRNAs are defined as endogenous 22 nt RNAs that play important regulatory roles in animals and plants by binding to the 3′ untranslated regions (UTRs) of target mRNAs, causing translation to be blocked and/or mRNA degradation [2,3]. miRNAs play diverse roles in carcinogenesis involved in the regulation of tumor proliferation, invasion, apoptosis and therapy resistance, and may act as oncogenes or tumor suppressors depending on the target mRNAs [4-6]. High-throughput technologies such as microarrays and next generation sequencing have showed global expression pattern of miRNAs, and quite a number of miRNAs which are dysregulated in several malignancies including CRC may act as novel oncogenes or tumor suppressor genes [7,8]. However, the mechanism underlying CRC tumorigenesis was not clear enough for the lacking of target genes information.

miR-378-5p is reported dysregulated expressed in several malignancies and the function of miR-378-5p is complicated because it can be oncogenic in glioblastoma, breast cancer and non-small cell lung cancer [9-11] or a tumor suppressor in liver cancer, gastric cancer and oral cancer [12-14]. So, identification of cancer-specific miRNAs targets is critical for understanding their roles in tumorigenesis, and may be important for finding out novel prognostic and therapeutic targets. Several studies have reported that miR-378-5p was significantly down-regulated in CRC [15-18]. However, the mechanism of miRNA-378-5p in CRC development is not very clear for poor targeting information.

In the present study, we identify that miR-378-5p is down-regulated in CRC and can suppress cell proliferation and induce apoptosis in CRC cells. We prove the potential tumor suppressor role of miR-378-5p involved in CRC by identifying one new targeting gene BRAF. Furthermore, we show that miR-378-5p suppress cell proliferation and induce apoptosis in CRC cells through RAS/RAF/MEK/ERK pathway. Our data may suggest a new perspective on how miR-378-5p involved in colon cancer.

## Materials and methods

### Human tissue specimens

47 paired CRC and normal tissues were collected from the Department of General Surgery, Rui Jin Hospital, Shanghai, China. CRC tissues were obtained from patients undergoing resection, and adjacent colon tissues were obtained from distal normal colon tissue of colon cancers. Informed consent was obtained from each patients and the study was approved by the Ethics Committee of Shanghai Jiao Tong University, Shanghai, China. All tissues were frozened to −80°C for subsequent experiments. All clinic pathologic and biological data were available for those patients. Patients’ characteristics of clinical-pathologic features were listed in Table 1. Additionally, five normal colorectal tissues were obtained from health people by colonoscopy and exclude colorectal cancer and polyps.

**Table 1:** **Patients’ characteristics of clinical-pathologic features**

**Characteristics**	**No. of patients (n = 47)**	**Percent (%)**
Age at diagnosis (year)		
≤60	17	36
>60	30	64
Sex		
Male	23	49
Female	24	51
Tumor size (cm)		
≤5	35	74
>5	12	26
Histological grading		
Well, moderate	38	81
Poor, mucinous	9	19
Depth of invasion		
T_1_-T_2_	7	15
T_1_-T_2_	40	85
Lymph node metastasis		
Negative	26	53
Postive	21	47
Clinicopathological staging		
I	2	4
II	23	49
III	15	32
IV	7	15
Location		
Colon	26	55
Rectum	21	45

### Cell lines

Human CRC cell lines LoVo, CaCo2, SW1116, SW480, HCT-116 and human embryonic kidney cell line HEK 293 T were purchased from the Cell Bank of the Chinese Academy of Sciences (Shanghai, China). The cells were incubated in RPMI1640 (PAN Biotech, Aidenbach, Germany) with 10% FBS (PAA Laboratories, Pasching, Australia) except for HEK 293 T which was incubated in Dulbecco’s modified Eagle’s medium (DMEM) (PAN Biotech) with 10% FBS (PAA Laboratories). All cells were cultured at 37°C in an atmosphere of 5% CO2.

### Quantitative reverse transcription-polymerase chain reaction

Total RNA of 47 paired CRC and normal tissues and cells were isolated using TRIzol reagent according to the manufacturer’s instructions (Invitrogen, California, USA). The concentration of all RNAs was measured using spectrophotometer (Thermo Fisher Scientific Inc., Waltham, USA) and 1 μg RNA was used for complementary DNA (cDNA) synthesis using the ReverTra Ace-α- (TOYOBO, Osaka, Japan) according to the manufacturer’s instructions. Real-time PCR was performed using the SYBR® regent (TOYOBO, Kita-ku, Osaka, Japan). The reverse transcription primer of miR-378-5p was 5′-GTC GTA TCC AGT GCA GGG TCC GAG GTA TTC GCA CTG GAT ACG ACA CAC A-3′ (stem-loop primer) and the amplification primers were as follows: 5′- GC CTC CTG ACT CCA GGT CC-3′ (sense) and 5′- GTG CAG GGT CCG AGG T-3′ (antisense). Primers for amplification of control U6 small nuclear RNA were: 5′-CTC GCT TCG GCA GCA CA-3′ (sense) and 5′-AAC GCT TCA CGA ATT TGC GT-3′ (antisense). Primers for BRAF were: 5′-CTT CCC CAG ACC GCG ATT C-3′ (sense) and 5′-CGA CCA CCT CTA TGG TGA CCT-3′ (antisense). Primers for β-actin were: 5′- AGC AGC ATC GCC CCA AAG TT-3′ (sense) and 5′-GGG CAC GAA GGC TCA TCA TT-3′ (antisense). Amplification was performed using Light Cycler 480II (Roche, Basel, Switzerland) and the amplification procedure consisted of 40 cycles (95°C for 10 seconds, 55°C for 10 seconds, 72°C for 20 seconds) following an initial denaturation at 95°C for 20 seconds. The fold change in target mRNA or miRNA expression was caculated using the 2^-ΔΔCt^ method following normalizatoin to β-actin or U6 expression respectively.

### RNA interference

BRAF and control siRNA were purchased from GenePharma company. The sequences of BRAF siRNA was: 5′-GAU GGC GGC GCU GAG CGG UdTdT-3′ (sense) and 5′-ACC GCU CAG CGC CGC CAU CdTdT-3′ (antisense), negative control siRNA was: 5′-UUC UCC GAA CGU GUC ACG UdTdT-3′ (sense), 5′-ACG UGA CAC GUU CGU AGA AdTdT-3′ (antisense). BRAF and control siRNA were transfected to CRC cell lines SW480 and HCT-116 in a final concentration of 20 nM using transfection reagent INTERFERin™ (Polyplus, Berkeley, CA, USA). 48 hours after transfection, cells were harvested for qRT-PCR, cell cycle and cell apoptosis analysis while western blot analysis was performed after 72 hours.

### Western blot analysis

Cells were harvested and lysed using Cell Lysis Buffer (Cell Signaling Technology, Danvers, MA, USA) and separated by 10% SDS-polyacrylamide gel (SDS-PAGE) and blotted to polyvinylidene fluoride (PVDF) membranes (Millipore, Darmstadt, Germany). After blocking with 5% non-fat milk in TBST (1‰), the PVDF membranes were incubated overnight at 4°C with anti-BRAF (#9433), anti-phosphorylated (p-)ERK (#9106) (both from Cell Signaling Technology; both diluted 1:1000) and anti-c-Myc (sc-40), anti-Bcl2 (sc-7382) (both from Santa Cruz Biotechnology Inc., California, USA; both diluted 1:1000) and anti-β-actin (A5441) (Sigma, St Louis, MO, USA), followed by incubation with a horseradish peroxidase-conjugated secondary antibody (diluted 1:2000) for 1 hours at room temperature. Protein bands were detected using Chemiluminescent Western Blot Scanner (Gene Company, HongKong, China). The β-actin band intensity served as the control for BRAF, p-ERK, c-Myc and Bcl2 expression.

### Vector construction and luciferase reporter assay

Wild-type (WT) or mutant (Mut) BRAF mRNA fragment was amplified and cloned into pMIR-Report construct (Ambion, Austin, TX, USA). Primers used for the BRAF WT mRNA fragment were: 5′-CCC AAG CTT AGG ACC TCA GCG AGA AAG GAA GTC AT-3′ (sense) and 5′-CTA GAC TAG TAC ATC ACC ATG CCA CTT TCC CTT G-3′ (antisense). Primers used for the BRAF Mut mRNA fragment were: 5′-CCC AAG CTT ACC TGG ACA GCC TCA AAG GAA GTC AT-3′ (sense) and 5′-CTA GAC TAG TAC ATC ACC ATG CCA CTT TCC CTT G-3′ (antisense). The vectors were verified by direct sequencing. HEK 293 T cells were placed onto 24-well plate and co-transfected with pMIR-BRAF-WT mRNA reporter plasmids (100 ng) or pMIR-BRAF-Mut mRNA reporter plasmids (100 ng), pMIR-TK (25 ng) and miR-378-5p mimics or negative control oligonucleotides (50 nM) using jetPEI (Polyplus). After 24 hours, cells were harvested and the reporter activity was detected using Dual-luciferase reporter assay system (Promega, Madison, Wisconsin, USA).

### Cell proliferation and cell cycle analysis

SW480 and HCT-116 cells were transfected with miR-378-5p mimics or negative control oligonucleotides using INTERFERin (Polyplus). Cell proliferation was detected at 24 hours, 48 hours, 72 hours and 96 hours after transfection using Cell Counting Kit-8 (Dojindo, Kumamoto, Japan) according to the manufacturer’s instructions. In short, 1 × 10^4^ cells were seeded onto 96-well plates per well in a final volume of 100 μl. At the indicated time, 10 μl CCK-8 solution was added to each well and cells were incubated at 37°C for 30 minutes before detecting the absorbance at 450 nm. For cell cycle assay, 48 hours after the SW480 and HCT-116 cells were transfected, the culture medium was changed to RPMI-1640 without serum for 24 hours, followed by another 6 hours cultured in RPMI-1640 with 10% FBS. Then the cells were harvested and fixed in 70% ethanol at 4°C overnight. Cells were stained with propidium iodide (PI) (Biolegend, California, USA) solution at a final concentration of 50 μg/ml which containing 50 μg/ml RNase A. Cell cycles were analyzed by flow cytometry (BD LSRII, San Jose, CA, USA).

### Cell apoptosis analysis

Cell apoptosis analysis was performed using phycoerythrin (PE)-annexinV apoptosis detection kit (BD PharMingen, San Jose, CA, USA). For cell apoptosis analysis, cells were seeded in 6-well plates at 8 × 10^5^ per well. Seventy-two hours after transfection, cells in the suspension and that were adhered were harvested and labeled with AnnexinV for 15 minutes in dark place. 50 μg/ml PI was added to each sample before the cell apoptosis distribution was analyzed by flow cytometry (BD LSRII, San Jose, CA, USA).

### Statistical analysis

All statistical analyses were carried out using the SPSS 16.0 statistical software package. Continuous variables were expressed as mean ± SEM. Differences between groups were calculated with Student’s t test. A two-tailed *P* value test was used with a *P* value of < 0.05 considered statistically significant.

## Results

### Expression of miR-378-5p is greatly decreased in CRC

**Figure 1:**
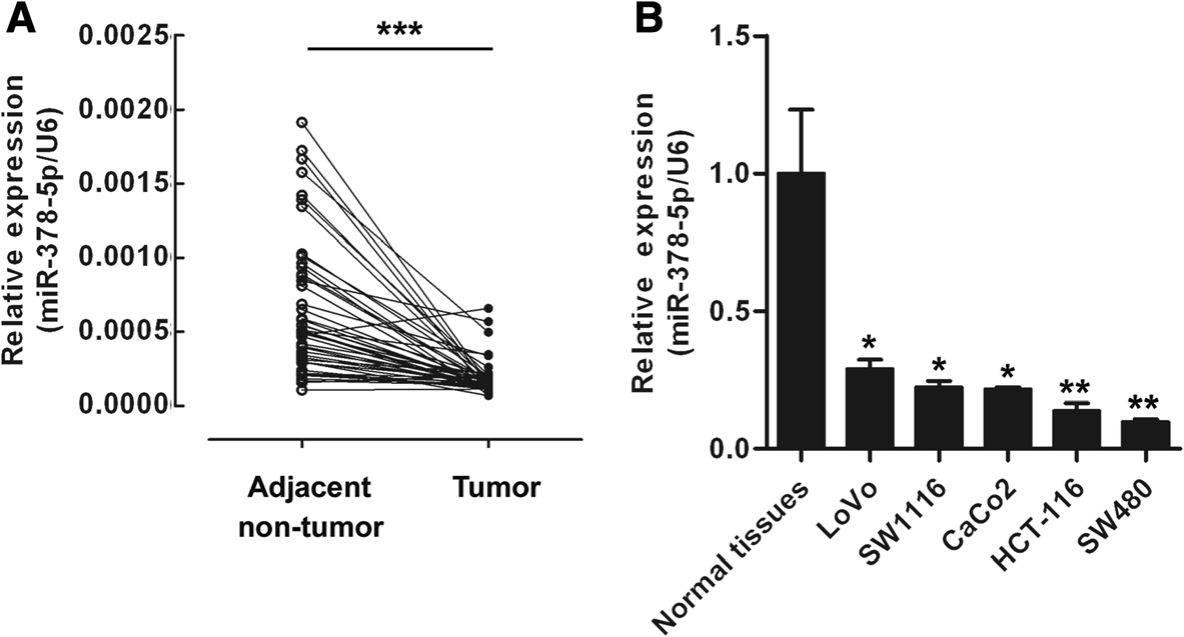
miR-378-5p is down-regulated in CRC samples and cell lines. **(A)** Expression of miR-378-5p was down-regulated in 47 CRC compared with adjacent non-tumor tissues. **(B)** The expression level of miR-378-5p was down-regulated in 5 CRC cell lines compared with normal tissues. *p <0.05, **p <0.01.

In order to confirm the involvement of miR-378-5p in CRC, we tested the relative expression level of miR-378-5p in 47 CRC tissues and corresponding adjacent non-tumor tissues using qRT-PCR. The results indicated that miR-378-5p was greatly decreased in CRC tissues compared with adjacent non-tumor tissues (44/47, 93.6%, *p* <0.001) (Figure 1A). It was also shown that miR-378-5p was down-regulated in 5 CRC cell lines, compared with 5 normal colorectal tissues (Figure 1B). These observations suggest that miR-378-5p may be a tumor suppressor in CRC.

### miR-378-5p inhibits proliferation of CRC cells in vitro

**Figure 2:**
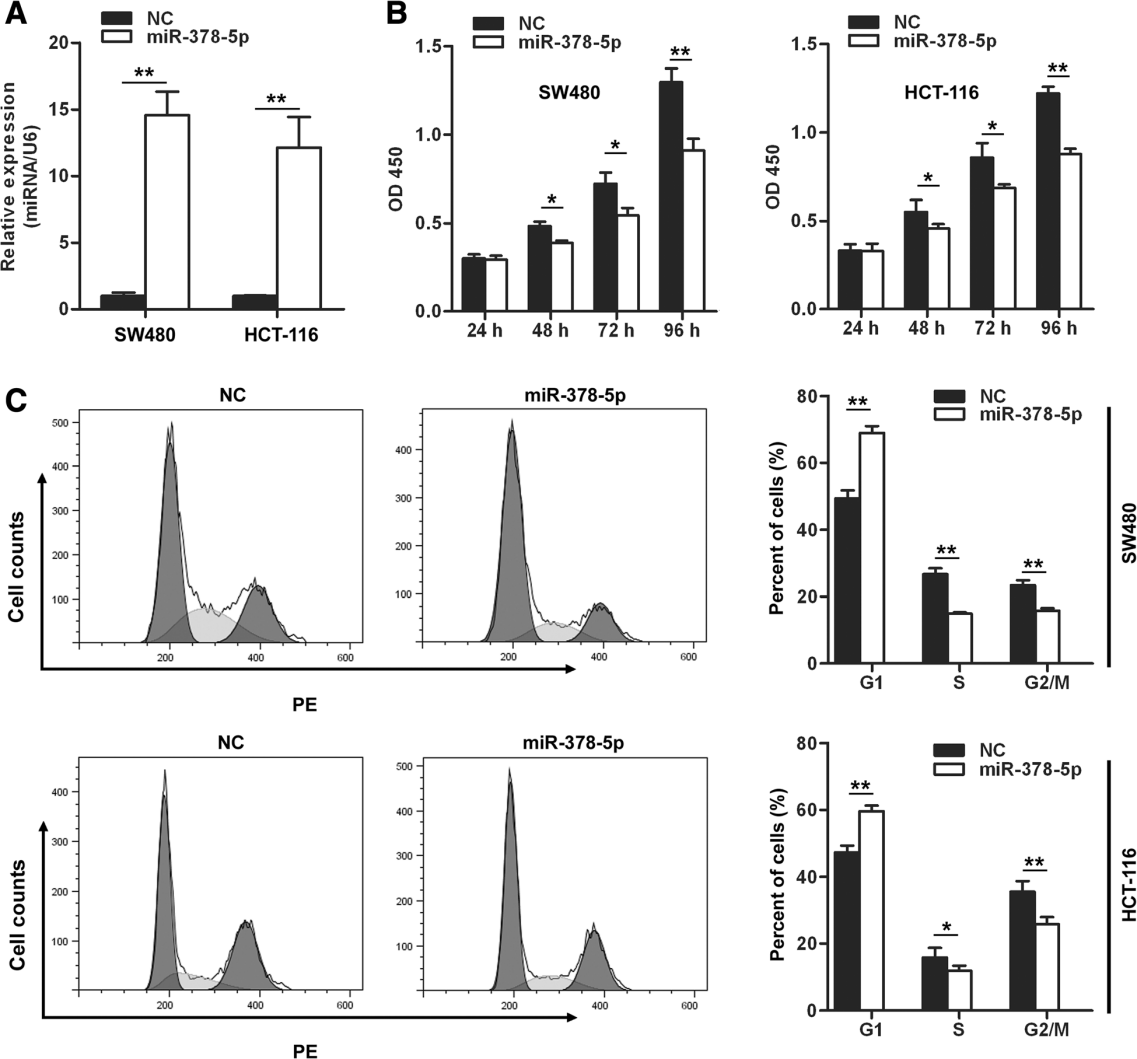
miR-378-5p overexpression inhibits CRC cells growth. **(A)** miR-378-5p mimics restored miR-378-5p expression in both SW480 and HCT-116 cells. **(B)** Overexpression of miR-378-5p in CRC cells suppressed cell proliferation. **(C)** Overexpression of miR-378-5p in CRC cells blocked G1/S transition. *p <0.05, **p <0.01. NC, negative control oligonucleotides.

The decreased expression of miR-378-5p in CRC tissues inspired us to assume miR-378-5p to be a tumor suppressor. The expression level of miR-378-5p was decreased in CRC cell lines (Figure 1B) and transfection of miR-378-5p mimics restored its expression level in both SW480 and HCT-116 CRC cells (Figure 2A). Then we investigated the effects of miR-378-5p restoration on these two cell lines. miR-378-5p mimics and negative control oligonucleotides were transfected into SW480 and HCT-116 cells and proliferation was tested by CCK-8 assay. As shown in Figure 2B, proliferation of CRC cells was suppressed following transfection with miR-378-5p at 48 hours (19.5%, *p* <0.05), 72 hours (24.6%, *p* <0.05) and 96 hours (29.8%, *p* <0.01) in SW480 cells and 48 hours (16.8%, *p* <0.05), 72 hours (26.1%, *p* <0.05) and 96 hours (28.1%, *p* <0.01) in HCT-116 cells. Taken together, the results indicate that miR-378-5p inhibits the proliferation of CRC cells *in vitro*.

Decreased cell proliferation in cancer cells relates closely to cell cycle arrest. We then detected whether cell cycle arrest contributed to the growth inhibition of miR-378-5p transfected cells. The result showed that the cell cycle was arrested in G1 phase, with 68.9% of miR-378-5p transfected cells in G0/G1 versus 49.4% of control cells in SW480 cells. Similar effects of miR-378-5p were found in HCT-116 cells, with 59.6% of miR-378-5p transfected cells in G0/G1 versus 47.2% of control cells (Figure 2C). These results demonstrate that overexpression of miR-378-5p inhibits growth of CRC cells by blocking G1/S transition.

### miR-378-5p induces apoptosis of CRC cells in vitro

**Figure 3:**
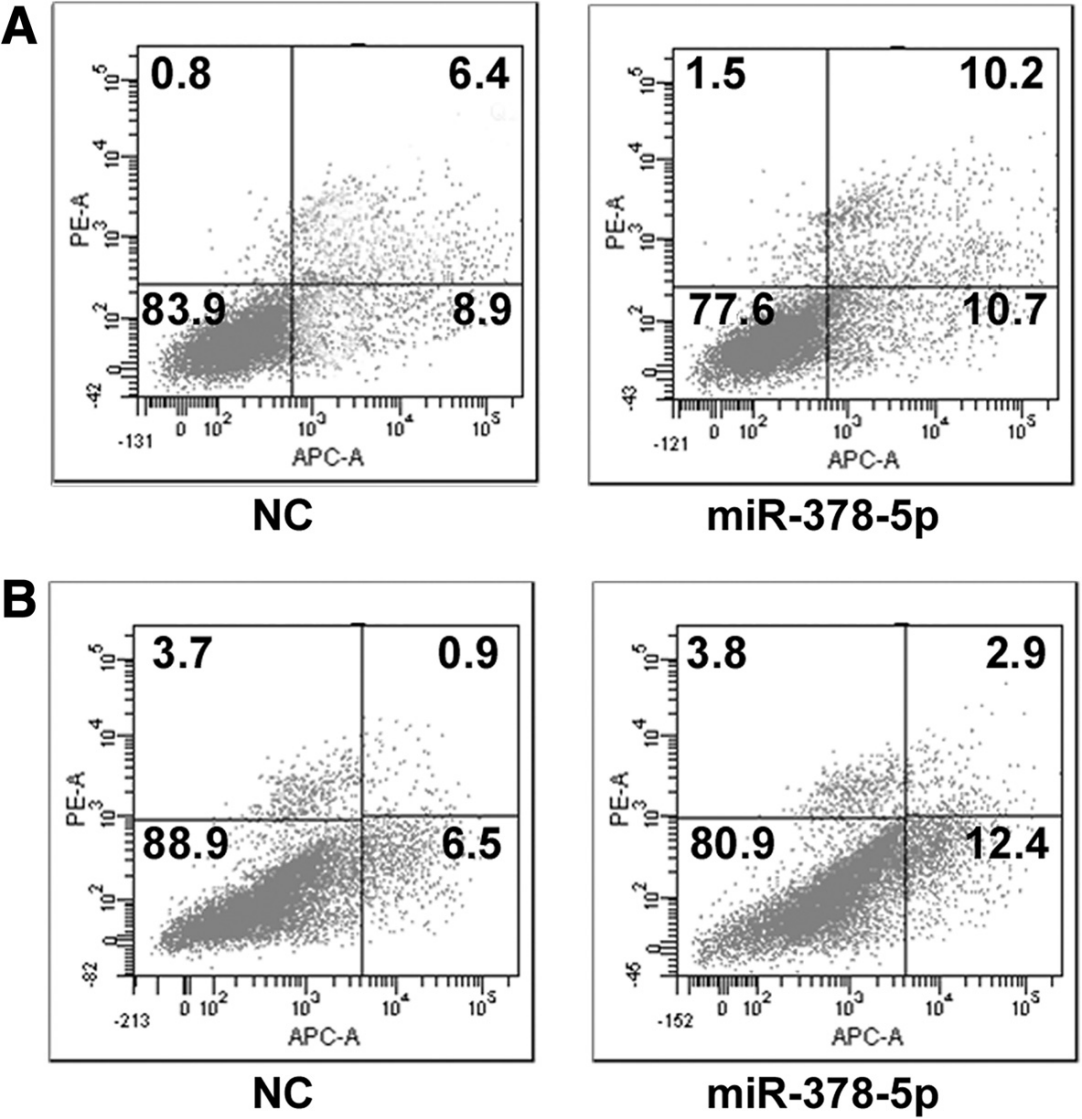
miR-378-5p overexpression induces CRC cells apoptosis. Cell apoptosis was analyzed by FACS analysis. miR-378-5p mimics induced CRC cells apoptosis in SW480 **(A)** and HCT-116 **(B)**.

Next, the ability of miR-378-5p to induce apoptosis in CRC cell lines was evaluated by co-staining with AnnexinV and propidium iodide (PI). miR-378-5p mimics and negative control oligonucleotides were transfected into SW480 and HCT-116 cells, then apoptosis was tested by flow cytometric assay. The staining demonstrated that miR-378-5p could significantly induce apoptosis in SW480 and HCT-116 cells compared with the negative control groups with a concomitant decrease in the viable cell population (Figure 3A,B). This points to a proapoptotic role of miR-378-5p and suggests that miR-378-5p affects apoptotic pathways in regulating tumorigenicity.

### miR-378-5p directly targets BRAF in CRC cells

**Figure 4:**
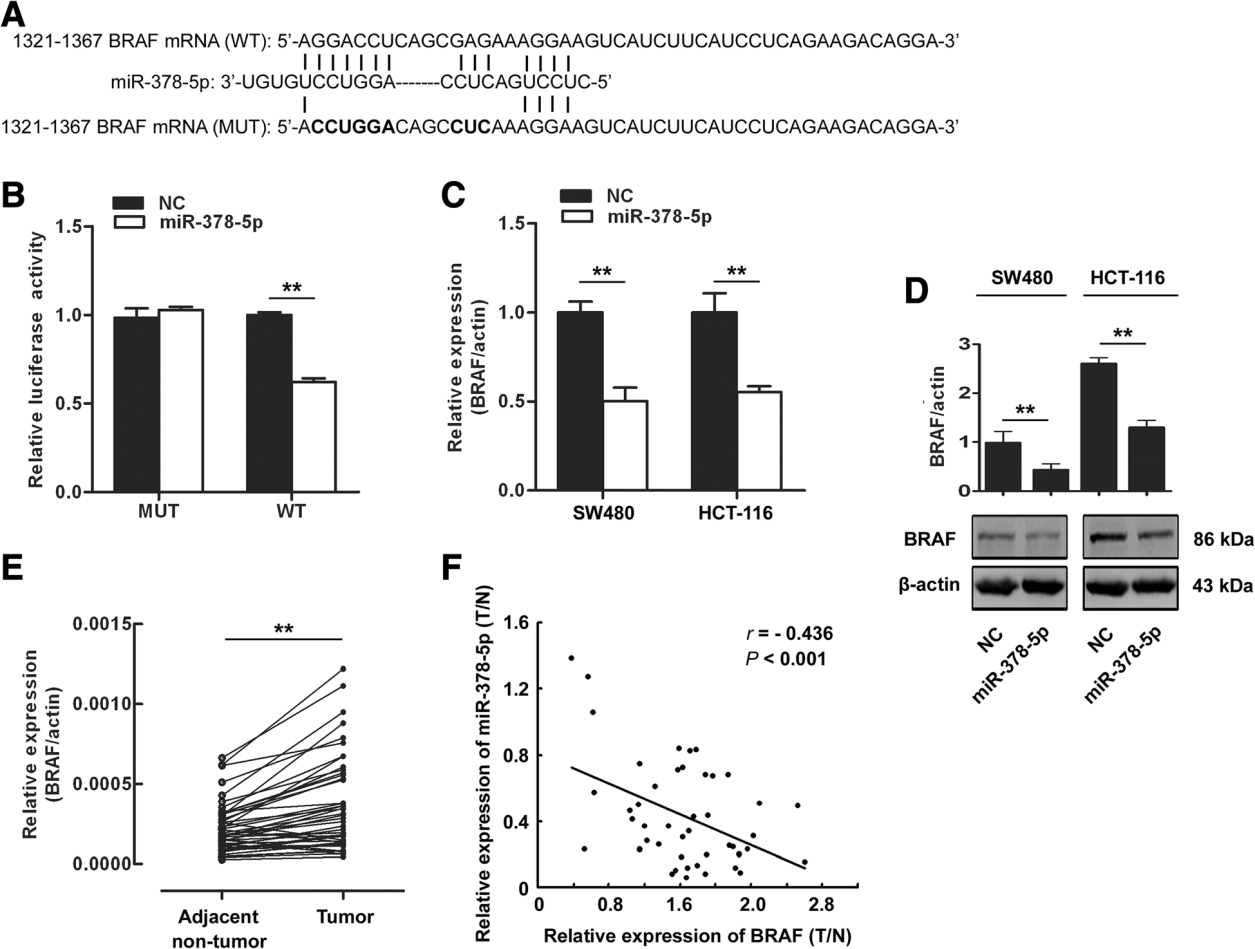
miR-378-5p down-regulates BRAF expression in human CRC cells. **(A)** Wild-type (WT) and mutant (Mut) of putative miR-378-5p targeting sequences in BRAF mRNA. Mutant sequences were shown in bold type. **(B)** miR-378-5p mimics inhibited wild-type but not mutant BRAF reporter activity in HEK 293 T cells. **(C)** miR-378-5p mimics down-regulated the endogenous BRAF mRNA levels in CRC cells. **(D)** miR-378-5p down-regulated the endogenous BRAF protein level in CRC cells. **(E)** Expression of BRAF was up-regulated in 47 CRC compared to adjacent non-tumor tissues. **(F)** A negative Spearman correlation between miR-378-5p and BRAF mRNA was found in 47 CRC tissues. *p <0.05, **p <0.01. NC, negative control oligonucleotides.

In order to investigate the underlying mechanisms of miR-378-5p in CRC, biological function of CRC pathogenesis-related genes was further analyzed. In 2013, Aleksandra Helwak and his colleagues identified more than 18000 high-confidence miRNA-mRNA interactions in HEK 293 cells by CLASH [19]. However, whether these miRNA:mRNA pairings also exit in CRC cells remaining to be explored. The CLASH data have showed that 103 genes were targeted by miR-378-5p. Among these genes, we focus on gene BRAF, a central component of the RAS/RAF/MEK/ERK pathway, which is involved in many cellular processes including cell growth, apoptosis and other cellular functions [20]. From the CLASH data in HEK 293 cells, the potential targeting sequence for miR-378-5p with a calculated energy of −17.1 kcal/mol is within the protein coding region of BRAF mRNA from 1321 to 1367. To verify the interaction of miR-378-5p with BRAF, we first performed luciferase reporter assays in HEK 293 T cells. We cloned the potential targeting sequence of miR-378-5p into a luciferase reporter vector. As shown in Figure 4B, transfection of miR-378-5p caused a significant decrease in luciferase activity in cells transfected with the reporter plasmid with wild-type targeting sequence of BRAF mRNA but not reporter plasmid with mutant sequence. Then, we explored whether the endogenous BRAF in CRC cells was regulated similarly. The mRNA and protein level of BRAF in SW480 and HCT-116 cells was also analyzed after transfection of miR-378-5p. As shown in Figure 4C and D, The level of BRAF mRNA and protein was consistently and substantially down-regulated by miR-378-5p. This result indicates that miR-378-5p can bind directly to BRAF and inhibits the expression of BRAF. Knowing BRAF was the target of miR-378-5p, we tested the expression of BRAF in the 47 CRC and adjacent non-tumor tissues. The results indicated that the expression level of BRAF mRNA was greatly increased in CRC comparing to adjacent non-tumor tissues (42/47, 89.4%, *p* <0.001) (Figure 4E) and was inversely related to the expression of miR-378-5p (Figure 4F).

### miR-378-5p inhibits the proliferation and induces apoptosis of CRC cells via regulation of RAS/RAF/MEK/ERK pathway

**Figure 5:**
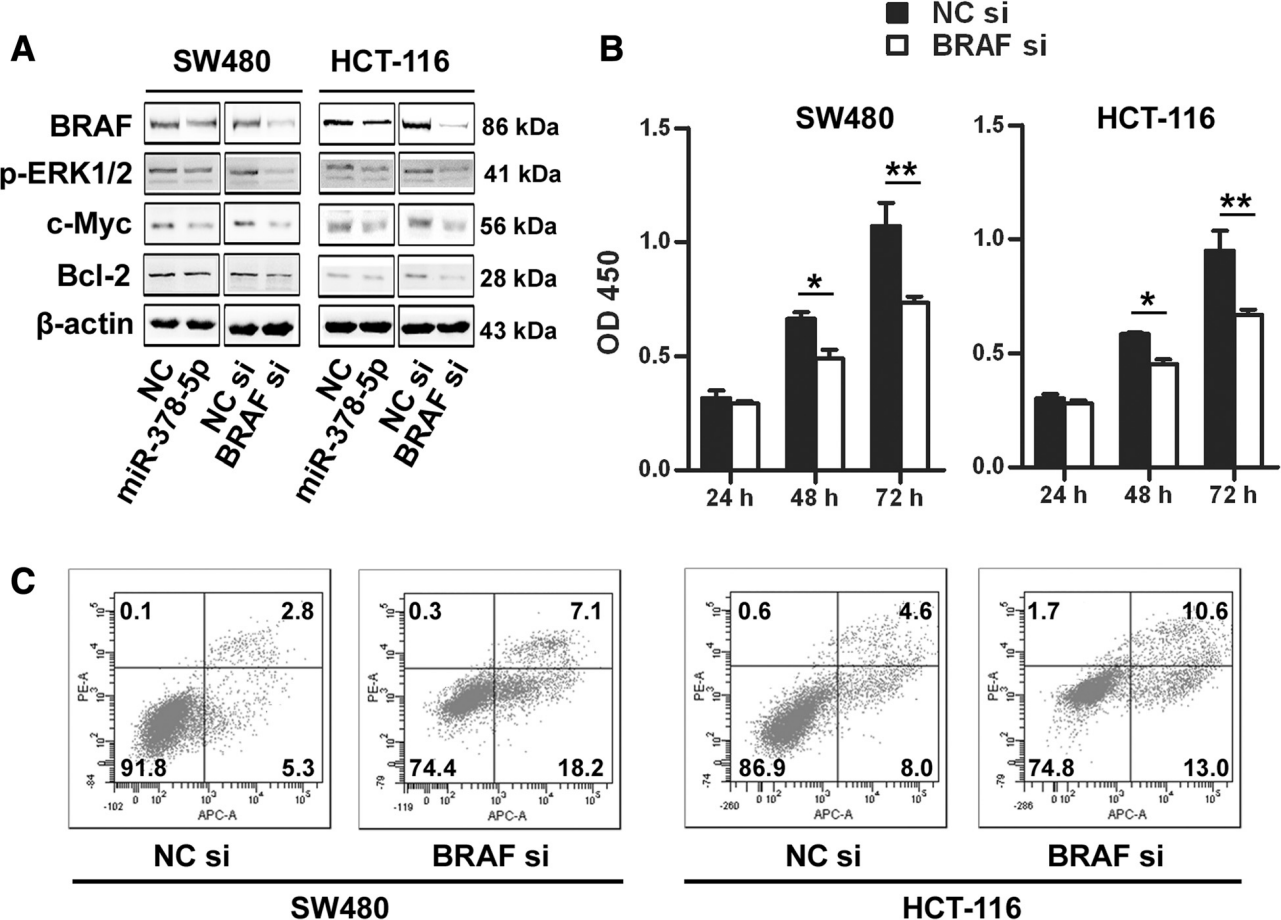
Knockdown of BRAF significantly inhibits cell proliferation and induces apoptosis in CRC cells. **(A)** Down-regulation of BRAF by miR-378-5p mimics and knockdown of BRAF by siRNA significantly inhibited RAS/RAF/MEK/ERK pathway in CRC cells. **(B)** Knockdown of BRAF by siRNA significantly inhibited proliferation in CRC cells. **(C)** Knockdown of BRAF by siRNA significantly induced apoptosis in CRC cells. **p <0.01. NC, negative control.

Since overexpression of miR-378-5p suppressed proliferation and induced apoptosis of CRC cells, and given that BRAF is a direct target of miR-378-5p, we hypothesized that the inhibitory effect of miR-378-5p on CRC cell viability might be achieved via targeting BRAF. In order to investigate this hypothesis, we tested whether RNAi-mediated reduction in BRAF influence the cell growth just like miR-378-5p in CRC cells. BRAF is a central component of the RAS/RAF/MEK/ERK pathway and most BRAF targeting genes were involved in cell growth regulation (such as c-Myc) or apoptosis (such as Bcl-2) [21]. So we investigated effects of miR-378-5p mimics and BRAF RNAi on RAS/RAF/MEK/ERK pathway in CRC cells. SW480 and HCT-116 cells were transfected with miR-378-5p or BRAF siRNA, and protein levels of RAS/RAF/MEK/ERK pathway were examined b ywestern blot. The results showed that protein levels of BRAF, p-ERK, c-Myc and Bcl-2 were consistently down-regulated by both miR-378-5p mimics and BRAF siRNA (Figure 5A). In addition, treatment of cells with BRAF siRNA markedly suppressed cell proliferation at 48 hours (26.3%, *p* <0.05) and 72 hours (31.3%, *p* <0.01) in SW480 cells and 48 hours (22.7%, *p* <0.05) and 72 hours (29.6%, *p* <0.01) in HCT-116 cells (Figure 5B) and significantly induced apoptosis in SW480 and HCT-116 cells (Figure 5C). These findings suggest that miR-378-5p suppresses CRC cells growth and induces apoptosis, at least in part, by targeting BRAF.

## Discussion

In this study, we studied the expression profile of miR-378-5p in CRC. Our results showed that miR-378-5p was down-regulated in CRC tissues and cell lines which agreed with the previous studies [15-18]. In addition, we identified BRAF as a new direct and functional target of miR-378-5p in CRC cells. Overexpression of miR-378-5p in CRC cells significantly decreased the proliferation and induced apoptosis by regulating RAS/RAF/MEK/ERK pathway.

Since the first miRNA lin-4 has been discovered in 1993, about 1881 miRNA sequences have been found in human genome. By attaching to the sequence-complementary mRNA targets, miRNAs can regulate the expression of nearly 30% protein coding genes [22,23]. Recently, deregulation of miRNAs in cancers and their roles in tumorigenesis have been increasingly investigated [24-26]. Michael firstly completed the identification of the miRNA populations changes of colorectal cancer in 2003 [27]. Now, several deregulated miRNAs in CRC such as miR-451, miR-34 and miR-135 have been shown to regulate cell apoptosis, growth, invasion and migration [28-30]. In this study, we demonstrated that miR-378-5p is dramaticly down-regulated in CRC cell lines. This was further confirmed by testing the relative expression level of miR-378-5p in 47 CRC tissues and corresponding adjacent non-tumor tissues using qRT-PCR. This was consistent with that miR-378-5p was decreased in CRC and gastric cancer [12,31]. miR-378-5p was found to be an independent prognostic factor and could inhibit cell growth and invasion in CRC by targeting vimentin [32]. However, the role of miR-378-5p in CRC is not well known for the limitation of target gene information. Our results demonstrated that BRAF was another direct target gene of miR-378-5p in CRC cells. Overexpression of miR-378-5p in CRC cells decreased both mRNA and protein level of BRAF in CRC cells. Our results also showed that miR-378-5p was negatively correlated with BRAF mRNA in CRC tissues. In addition, Overexpression of miR-378-5p in CRC cells significantly decreased the proliferation and induced apoptosis by regulating RAS/RAF/MEK/ERK pathway. Interestingly, the function of miR-378-5p is complicated because it can be oncogenic in glioblastoma, breast cancer and renal cell carcinoma [9,10,33] or a tumor suppressor in liver cancer, colorectal cancer, gastric cancer and oral cancer [12,13,31]. Why miR-378-5p functions so differently in different kinds of tumors? This may owe to the differences of the tumor microenvironment, including the external stimula, the inflammational environment or the stroma cells, which lead miR-378-5p to exhibit opposite effects, but the mechanism needs further study.

BRAF is a member of the Raf family that are serine/threonine kinases and plays an important role in cell proliferation, differentiation and apoptosis by participating in the mitogen-activated protein kinase (MAPK)/extracellular signal-regulated kinase (ERK) signaling pathway [34-36]. It has been reported that 59% of melanomas, 18% of CRCs, 11% of gliomas, and 4% of lung adenocarcinomas and ovarian carcinomas contain BRAF mutations, which result in persistent activation of the MAPK/ERK pathway that leads to sustained proliferative signaling [37]. However, recent studies show that metastases of CRC rarely contain BRAF mutation with BRAF wild-type primary tumours [38]. The relationship between the presence of BRAF mutation and the effect of anti-EGFR monoclonal antibodies is argumentative.Some studies show that patients with BRAF mutation may have a poor reaction to vemurafenib which is an anti-EGFR monoclonal antibodies [39]. On the contrary,Troiani thought BRAF mutation had no necessary connection with the sensitivity to selumetinib [40]. Increasing studies revealed that deregulation of miRNAs was responsible for abnormal expression of human BRAF in many cancers [41]. Several miRNA were reported as regulators of BRAF in different cancers such as miR-524-5p in melanoma, miR-143 and miR-145 in CRC [42,43]. In this study, BRAF was identified to be directly targeted and regulated by miR-378-5p in CRC cells. Our results agreed with the viewpoint that a single mRNA molecule can been regulated by multiple miRNA genes in different cells [44-46].

In conclusion, our study demonstrates that the expression of miR-378-5p is decreased in CRC. miR-378-5p can inhibit proliferation of CRC cells and induce CRC cells apoptosis by directly suppressing the expression of BRAF. All of the above indicate that miR-378-5p is served as an anti-oncogene in CRC.

## Abbreviations

MiRNAs: MicroRNAs
CRC: Colorectal cancer
qRT-PCR: Quantitative reverse transcription-polymerase chain reaction
UTRs: Untranslated regions
FBS: Fetal Bovine serum
DMEM: Dulbecco’s modified eagle medium
SDS-PAGE: Dodecyl sulfate, sodium salt -polyacrylamide gel electrophoresis
WT: Wild-type
Mut: Mutant
PI: Propidium iodide
SEM: Standard error of the mean
CLASH: Cross-linking ligation and sequencing of hybrid
ERK: Extracellular signal-regulated kinase
MAPK: Mitogen-activated protein kinase

## References

[1] Meyerhardt JA, Mayer RJ (2005). Systemic therapy for colorectal cancer. N Engl J Med.

[2] Bartel DP (2004). MicroRNAs: genomics, biogenesis, mechanism, and function. Cell.

[3] He L, Hannon GJ (2004). MicroRNAs: small RNAs with a big role in gene regulation. Nat Rev Genet.

[4] Esquela-Kerscher A, Slack FJ (2006). Oncomirs - microRNAs with a role in cancer. Nat Rev Cancer.

[5] Cho WC (2007). OncomiRs: the discovery and progress of microRNAs in cancers. Mol Cancer.

[6] Manikandan J, Aarthi JJ, Kumar SD, Pushparaj PN (2008). Oncomirs: the potential role of non-coding microRNAs in understanding cancer. Bioinformation.

[7] Visone R, Croce CM (2009). MiRNAs and cancer. Am J Pathol.

[8] Wu WK, Lee CW, Cho CH, Fan D, Wu K, Yu J (2010). MicroRNA dysregulation in gastric cancer: a new player enters the game. Oncogene.

[9] Eichner LJ, Perry MC, Dufour CR, Bertos N, Park M, St-Pierre J (2010). miR-378(*) mediates metabolic shift in breast cancer cells via the PGC-1beta/ERRgamma transcriptional pathway. Cell Metab.

[10] Lee DY, Deng Z, Wang CH, Yang BB (2007). MicroRNA-378 promotes cell survival, tumor growth, and angiogenesis by targeting SuFu and Fus-1 expression. Proc Natl Acad Sci U S A.

[11] Chen LT, Xu SD, Xu H, Zhang JF, Ning JF, Wang SF (2012). MicroRNA-378 is associated with non-small cell lung cancer brain metastasis by promoting cell migration, invasion and tumor angiogenesi**s**. Med Oncol.

[12] Deng H, Guo Y, Song H, Xiao B, Sun W, Liu Z (2013). MicroRNA-195 and microRNA-378 mediate tumor growth suppression by epigenetical regulation in gastric cancer. Gene.

[13] Scapoli L, Palmieri A, Lo Muzio L, Pezzetti F, Rubini C, Girardi A (2010). MicroRNA expression profiling of oral carcinoma identifies new markers of tumor progression. Int J Immunopathol Pharmacol.

[14] Li LH, Gao Q, Wang XY, Guo ZJ (2013). miR-378 suppresses HBV-related hepatocellular carcinoma tumor growth by directly targeting the insulin-like growth factor 1 receptor. Zhonghua Gan Zang Bing Za Zhi.

[15] Callari M, Dugo M, Musella V, Marchesi E, Chiorino G, Grand MM (2012). Comparison of microarray platforms for measuring differential microRNA expression in paired normal/cancer colon tissues. PLoS One.

[16] Mosakhani N, Sarhadi VK, Borze I, Karjalainen-Lindsberg ML, Sundstrom J, Ristamaki R (2012). MicroRNA profiling differentiates colorectal cancer according to KRAS status. Genes Chromosomes Cancer.

[17] Faltejskova P, Svoboda M, Srutova K, Mlcochova J, Besse A, Nekvindova J (2012). Identification and functional screening of microRNAs highly deregulated in colorectal cancer. J Cell Mol Med.

[18] Wang YX, Zhang XY, Zhang BF, Yang CQ, Chen XM, Gao HJ (2010). Initial study of microRNA expression profiles of colonic cancer without lymph node metastasis. J Dig Dis.

[19] Helwak A, Kudla G, Dudnakova T, Tollervey D (2013). Mapping the human miRNA interactome by CLASH reveals frequent noncanonical binding. Cell.

[20] Wajapeyee N, Serra RW, Zhu X, Mahalingam M, Green MR (2008). Oncogenic BRAF induces senescence and apoptosis through pathways mediated by the secreted protein IGFBP7. Cell.

[21] Ciuffreda L, Del Bufalo D, Desideri M, Di Sanza C, Stoppacciaro A, Ricciardi MR (2009). Growth-inhibitory and antiangiogenic activity of the MEK inhibitor PD0325901 in malignant melanoma with or without BRAF mutations. Neoplasia.

[22] Hwang HW, Mendell JT (2006). MicroRNAs in cell proliferation, cell death, and tumorigenesis. Br J Cancer.

[23] Lewis BP, Burge CB, Bartel DP (2005). Conserved seed pairing, often flanked by adenosines, indicates that thousands of human genes are microRNA targets. Cell.

[24] Espinosa-Parrilla Y, Munoz X, Bonet C, Garcia N, Vencesla A, Yiannakouris N (2014). Genetic association of gastric cancer with miRNA clusters including the cancer-related genes MIR29, MIR25, MIR93 and MIR106: results from the EPIC-EURGAST study. Int J Cancer.

[25] Huang J, Wu J, Li Y, Li X, Yang T, Yang Q (2014). Deregulation of serum MicroRNA expression is associated with cigarette smoking and lung cancer. BioMed Res Int.

[26] Cristobal I, Rincon R, Manso R, Carames C, Aguilera O, Madoz-Gurpide J (2014). Deregulation of miR-200b, miR-200c and miR-429 indicates its potential relevant role in patients with colorectal cancer liver metastasis. J Surg Oncol.

[27] Michael MZ SMOC, van Holst Pellekaan NG, Young GP, James RJ (2003). Reduced accumulation of specific microRNAs in colorectal neoplasia. Mol Cancer Res.

[28] Chen MB, Wei MX, Han JY, Wu XY, Li C, Wang J (2014). MicroRNA-451 regulates AMPK/mTORC1 signaling and fascin1 expression in HT-29 colorectal cancer. Cell Signal.

[29] Kim NH, Cha YH, Kang SE, Lee Y, Lee I, Cha SY (2013). P53 regulates nuclear GSK-3 levels through miR-34-mediated Axin2 suppression in colorectal cancer cells. Cell Cycle.

[30] Nagel R, le Sage C, Diosdado B, van der Waal M, Oude Vrielink JA, Bolijn A (2008). Regulation of the adenomatous polyposis coli gene by the miR-135 family in colorectal cancer. Cancer Res.

[31] Guo J, Miao Y, Xiao B, Huan R, Jiang Z, Meng D (2009). Differential expression of microRNA species in human gastric cancer versus non-tumorous tissues. J Gastroenterol Hepatol.

[32] Zhang GJ, Zhou H, Xiao HX, Li Y, Zhou T (2014). MiR-378 is an independent prognostic factor and inhibits cell growth and invasion in colorectal cancer. BMC Cancer.

[33] Redova M, Poprach A, Nekvindova J, Iliev R, Radova L, Lakomy R (2012). Circulating miR-378 and miR-451 in serum are potential biomarkers for renal cell carcinoma. J Transl Med.

[34] Mikami M, Nosho K, Yamamoto H, Takahashi T, Maehata T, Taniguchi H (2006). Mutational analysis of beta-catenin and the RAS-RAF signalling pathway in early flat-type colorectal tumours. Eur J Cancer.

[35] Matsukuma S, Yoshihara M, Kasai F, Kato A, Yoshida A, Akaike M (2006). Rapid and simple detection of hot spot point mutations of epidermal growth factor receptor, BRAF, and NRAS in cancers using the loop-hybrid mobility shift assay. J Mol Diagn.

[36] Rodriguez-Viciana P, Tetsu O, Oda K, Okada J, Rauen K, McCormick F (2005). Cancer targets in the Ras pathway. Cold Spring Harb Symp Quant Biol.

[37] Davies H, Bignell GR, Cox C, Stephens P, Edkins S, Clegg S (2002). Mutations of the BRAF gene in human cancer. Nature.

[38] Santini D, Spoto C, Loupakis F, Vincenzi B, Silvestris N, Cremolini C (2010). High concordance of BRAF status between primary colorectal tumours and related metastatic sites: implications for clinical practice. Ann Oncol.

[39] Troiani T, Zappavigna S, Martinelli E, Addeo SR, Stiuso P, Ciardiello F (2013). Optimizing treatment of metastatic colorectal cancer patients with anti-EGFR antibodies: overcoming the mechanisms of cancer cell resistance. Expert Opin Biol Ther.

[40] Troiani T, Vecchione L, Martinelli E, Capasso A, Costantino S, Ciuffreda LP (2012). Intrinsic resistance to selumetinib, a selective inhibitor of MEK1/2, by cAMP-dependent protein kinase A activation in human lung and colorectal cancer cells. Br J Cancer.

[41] Nosho K, Igarashi H, Nojima M, Ito M, Maruyama R, Yoshii S (2014). Association of microRNA-31 with BRAF mutation, colorectal cancer survival and serrated pathway. Carcinogenesis.

[42] Liu SM, Lu J, Lee HC, Chung FH, Ma N (2014). miR-524-5p suppresses the growth of oncogenic BRAF melanoma by targeting BRAF and ERK2. Oncotarget.

[43] Pagliuca A, Valvo C, Fabrizi E, di Martino S, Biffoni M, Runci D (2013). Analysis of the combined action of miR-143 and miR-145 on oncogenic pathways in colorectal cancer cells reveals a coordinate program of gene repression. Oncogene.

[44] Wu GG, Li WH, He WG, Jiang N, Zhang GX, Chen W (2014). Mir-184 post-transcriptionally regulates SOX7 expression and promotes cell proliferation in human hepatocellular carcinoma. PLoS One.

[45] Ma Y, She XG, Ming YZ, Wan QQ. miR-24 promotes the proliferation and invasion of HCC cells by targeting SOX7. Tumour Biol. 2014. 10.1007/s13277-014-2018-6 25073511

[46] Shen F, Cai WS, Feng Z, Li JL, Chen JW, Cao J, et al. MiR-492 contributes to cell proliferation and cell cycle of human breast cancer cells by suppressing SOX7 expression. Tumour Biol. 2014. 10.1007/s13277-014-2794-z 25407488

